# Pilot study of the use of handheld 6-lead ECG for patients on acute general adult mental health wards who refuse traditional 12-lead ECG

**DOI:** 10.1192/bjo.2021.88

**Published:** 2021-06-18

**Authors:** Paul M Briley, Sudheer Lankappa

**Affiliations:** 1 Institute of Mental Health, University of Nottingham, Nottinghamshire Healthcare NHS Trust, Nottinghamshire Healthcare NHS Foundation Trust; 2 Institute of Mental Health, University of Nottingham, Nottinghamshire Healthcare NHS Foundation Trust

## Abstract

**Aims:**

To assess patient and clinician acceptability of handheld 6-lead ECG, for obtaining information about cardiac rhythm and electrical intervals, in acute general adult mental health ward inpatients who refuse traditional 12-lead ECG.

**Background:**

In a previous audit of patients admitted to four acute general adult mental health wards, we found that 1 in 4 patients refused 12-lead ECG for at least two weeks, with 1 in 6 refusing throughout their entire stay. ECG refusers were significantly more likely to have a psychotic illness than non-refusers and were thus more likely to benefit from medications that carry a risk of prolonging the QT interval. Less invasive, handheld, 6-lead ECG, which includes measurement of lead II (the lead used to define traditional QT-interval cut-off values) is available on the NHS supply chain. Whilst not providing the full range of information that 12-lead ECG is able to provide, handheld 6-lead ECG might be an acceptable alternative in patients who would otherwise never have any form of ECG performed.

**Method:**

We developed a Standard Operating Procedure for use of handheld 6-lead ECG and provided training for junior doctors on the four wards that were the subject of our original audit. These doctors were then able to offer the device to patients on their wards who refused 12-lead ECG. Doctors completed a short feedback form each time a handheld ECG was offered.

**Result:**

So far, handheld 6-lead ECGs have been offered to 17 patients who refused 12-lead ECGs. Mean age (± SD) was 36.1 (± 12.6) years, and 4 of these patients were female. 13 patients (76%) accepted a handheld ECG. One of these attempts failed due to patient agitation. Attempts took a mean of 7 (± 5.4) minutes. 54% of recordings were described as “very easy” by clinicians, whereas 15%, 23% and 8% were described as “somewhat easy”, “intermediate”, and “somewhat difficult”, respectively. Clinician difficulties focussed on patient movement with impact on electrode contact and trace quality. Where answered (N = 10), 90% of patients stated they would recommend a handheld ECG to others. Patients liked the speed of the process, that it felt “less scary”, and that it was less invasive and did not involve removing clothing.

**Conclusion:**

Our initial findings from this pilot suggest that handheld 6-lead ECG may be acceptable, both to clinicians and patients, as a means of obtaining information on cardiac rhythm and electrical intervals for patients who refuse 12-lead ECGs.

